# Scurvy: When it is a Forgotten Illness the Surgery Makes the Diagnosis

**DOI:** 10.2174/1874325001711011314

**Published:** 2017-11-20

**Authors:** Wajdi Bouaziz, Mohamed Ali Rebai, Mohamed Ali Rekik, Nabil Krid, Zoubaier Ellouz, Hassib Keskes

**Affiliations:** 1 Department of Orthopedic Surgery and Traumatology, Hbib Bourguiba University Hospital Sfax, Tunisia; 2 Marechal Leclerc Argentan Hospital, Argentan, France

**Keywords:** Scurvy, Vitamin C, Osteomyelitis, Subperiosteal hematoma, Musculoskeletal, Radiological

## Abstract

**Background:**

Unlike most of animal species, human beings lack the enzymatic process for the conversion of glucose to ascorbic acid (vitaminC), and therefore getting the vitamin from food sources is essential. The association of the various signs caused by a deficiency of vitamin C is called scurvy or Barlow’s disease, an easily treatable disease but can be fatal. It is rare in the developed countries and even economically underdeveloped societies in which the basic diet is already rich in ascorbate.

**Methods:**

We describe here the case of a 4-year-old girl with cerebral palsy, in whom diagnosis concerns were oriented for osteomyelitis, based upon clinical presentation, ultrasonic and magnetic resonance imaging, led to a surgery revealing subperiosteal hematomas that argues in favor of scurvy.

**Results:**

After vitamin C therapy, the symptoms are gone and the general condition of the patient improved despite persistent radiological signs.

**Conclusion:**

Recent studies of sporadic cases report a high incidence of scurvy in children with autism or psychomotor retardation and the fact that musculoskeletal manifestations are more common. The mosaics of the symptoms of scurvy are varied and include dermatological, dental, bone and systemic manifestations, making it a forgotten and misdiagnosed illness. A heightened awareness is needed to avoid an unnecessary surgery, unnecessary tests and procedures and to be able to start treatment for a potentially fatal but easily curable disease.

## 1 INTRODUCTION

Although infrequent, scurvy continues to be seen today within certain populations, such as, the elderly subjects, patients with neurological delay or psychiatric affections, or others with unusual alimentary diet.

Scurvy is a rare condition in pediatric patients, more seen in those with a developmental delay or autism, and some studies have found a correlation between infant scurvy and milk pasteurization which denatures vitamin C [1, 2]. Musculoskeletal manifestations are prominent in pediatric scurvy [3] and occur in 80% of patients [1, 4].

We can find non specific arthralgia, myalgia, hemarthrosis, muscular hemorrhage, and subperiosteal hematomas. These symptoms are more common in the lower extremities and the knee is the most affected joint [1].

The rarity and the polymorphisms of the clinical signs make scurvy an often unknown diagnosis [5].

Here we report a case of scurvy in a 4-year-old girl with cerebral palsy in whom initial concern for osteomyelitis, based upon clinical presentation, ultrasonic and magnetic resonance imaging, led to a surgery revealing subperiosteal hematomas.

## 2 CASE REPORT

A 4-year-old girl was admitted in the pediatrics department because of a bloody diarrhea and mild fever for one week. For the diagnosis of infectious colitis the patient was started on intravenous antibiotics.

The child was born full-term and weighed 2440 g with a context of acute fetal distress. She was diagnosed with central palsy, psyco-developmental delay, and epiliptogenic disorder put under valproic acid.

She was nonambulant with severe spastic paraplegia and incontinent of stool and urine.

Family history of allergy had been found with the mother with asthma.

There is no history of bone disease, or cancer. We had no idea about the father’s medical history.

Upon physical examination, the patient’s weight was less than the third percentile; she was also pale and has marked anxiety to strangers. She was febrile (38.8 °C), had a pulse of 124, blood pressure 100/64 mm Hg, and respiratory rate of 22.

Oral examination shows poor dental hygiene but the skin was normal.

One day after admission, right shoulder bruising and swelling were noticed with limited active motion’s range and pain on palpation of proximal humeral metaphysis. Full passive range of motion was noted. There were no abnormal findings on the rest of the musculoskeletal examination.

The biological samples showed the following results: white blood cell count: 10220/*μ*L, platelets count: 440000/*μ*L, hemoglobin: 5 g/dL (microcytic hypochromic aregenerative anemia), blood sedimentation rate >140 mm/h and a C - reactive protein of 90 mg/dL. A low level of iron was seen with a rate of 6micro-mole/l (11 to 27 micromole/l).

Hemostasis, alkaline phosphatase, calcium and phosphate levels were normal. A transfusion was necessary to raise the hemoglobin level to 9, 6 g/dL.

**Fig. (1):**
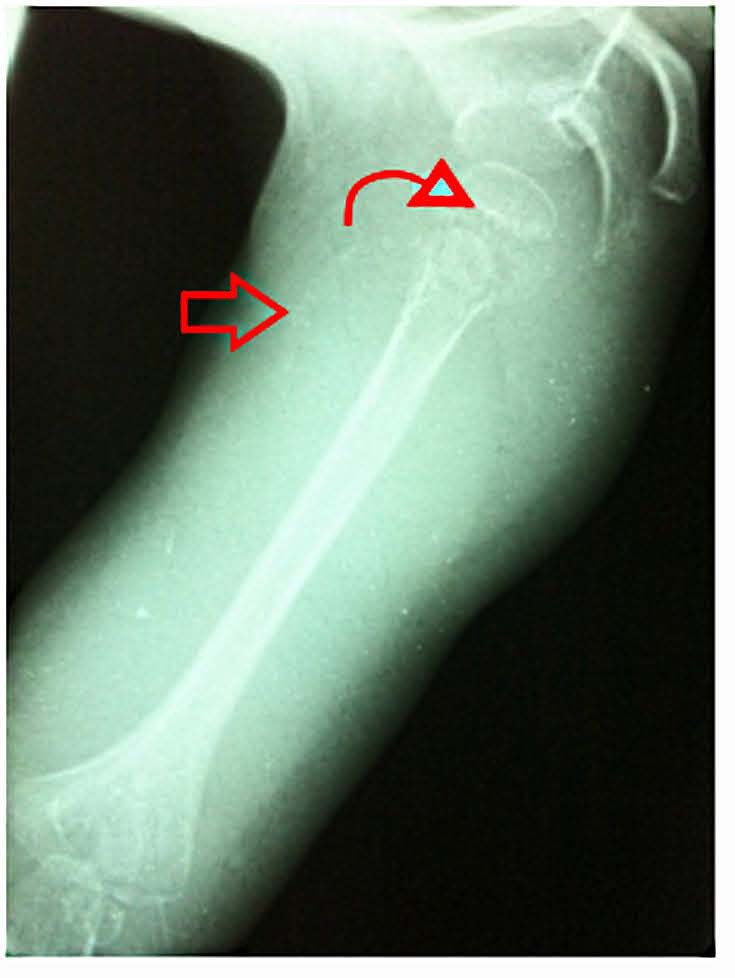
Radiograph of right shoulder shows dense, linear calcification in the distal metaphysis “white line of Frankel” (curved arrow) and periosteal separation (red arrow).

The coproculture done was negative. For the bloody diarrhea investigation, a proctoscopy was done revealing a solitary rectal ulcer which was attached to a thermometer-induced ulceration of the rectum. X rays of the right shoulder showed: osteopenia, an irregular thickened white line at the upper humeral metaphysis, a zone of rarefaction under the same metaphysis and a periosteal reaction (Fig. **1**).

**Fig. (2):**
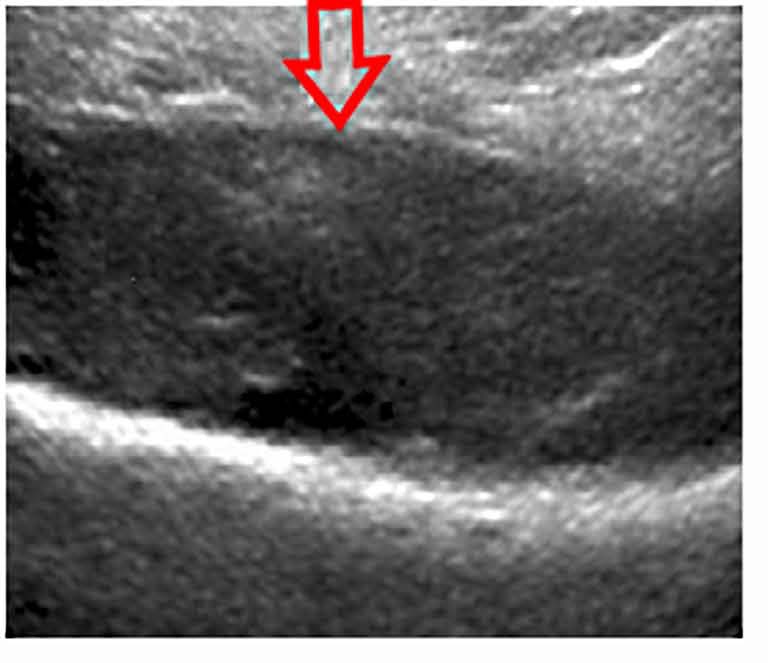
The sagittal ultrasound image shows heterogeneous hypoechoic lesions compatible with subperiosteal abscess (red arrow).

Ultrasound of the right shoulder showed infiltration and denseness of the soft tissues with detachment of the periosteum in the lateral face of the Humerus (Fig. **2**). Hence the diagnosis of subperiosteal abscess fistulized in soft tissues was suggested.

**Fig. (3):**
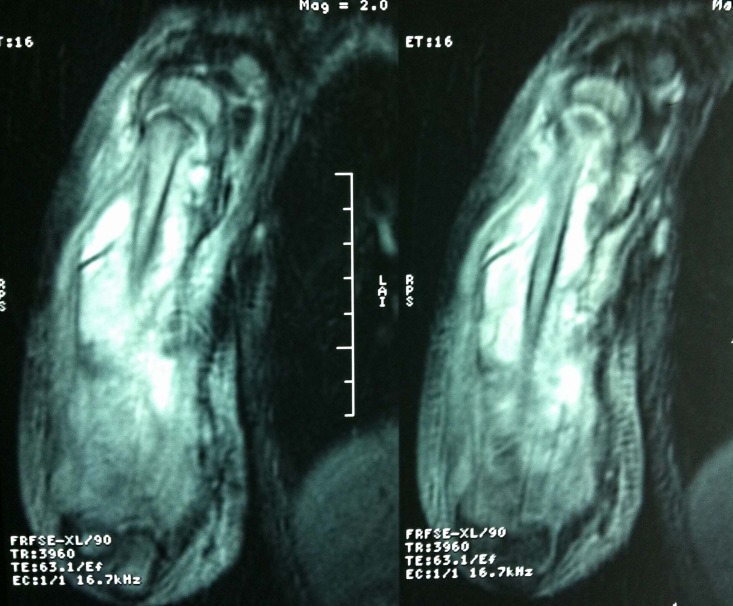
Shouldar MRI.

Shoulder MRI was performed and interpreted as osteomyelitis of the right humerus with fistulized subperiosteal abcess associated with an articular effusion of the elbow and shoulder joints (Fig. **3**).

Orthopedic surgeon was consulted for the concern of osteomyelitis.

**Fig. (4):**
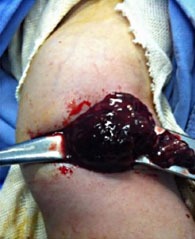
Needle insertion into the fluid collection yielding a gelatinous red material in the operating room.

**Fig. (5):**
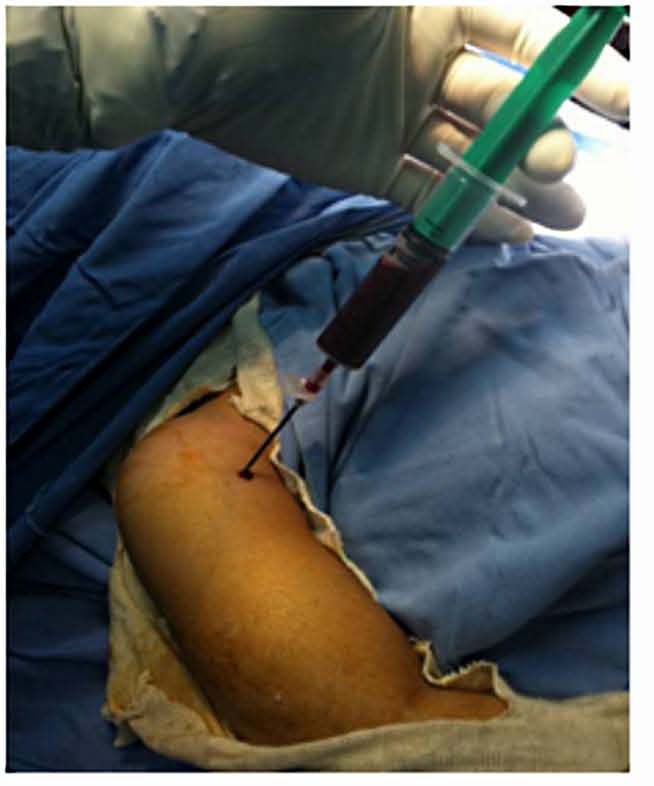
Subperiosteal hematomas evacuated after surgical approach.

A surgery was quickly considered and the aspiration of the collection by a needle bringing back a gelatinous red liquid (Fig. **4**). After surgical approach, it was clear that the collection interpreted as abscess was just a subperiosteal hematoma without any sign of infection (Fig. **5**).

Intravenous Oxacillin and Gentamycin were started for the concern of osteomyelitis. Bacterial cultures from the needle aspirate were negative and antibiotics have been stopped. The bone biopsy showed suggestive signs of a recent subperiosteal bleeding and abnormality of the collagen matrix, which was suggestive of scurvy.

In the absence of the technical board, the level of vitamin C was not available.

More detailed questioning was made in order to get an idea about the dietary habits. It shows that the patient was fed by his mother only with milk products.

The patient was placed under an oral supplementation of vitamin C at a dose of 250 mg per day. His mother was informed about dietary modification. Three weeks after vitamin C therapy, the child’s pain and the general condition of the patient improved.

## 3 DISCUSSION

Vitamin C is essential for humans and intimately concerned in the maintenance of intercellular connective tissue and stabilization of collagen triple helix [6, 7]. Since the human body is unable to synthesize vitamin C, its dietary intake must be with sufficient amounts [4, 7].

Collagen abnormalities, caused by lack of vitamin C explain the clinical manifestations of scurvy: stomatological deformations and the fall of teeth, vascular fragility causing bleeding and purpura, bone changes (in children) due to the inability of osteoblasts to produce the osteoid matrix, and skin changes related to the poor quality of keratin [7, 8].

Although, rare, scurvy in pediatric patients still appears. Scurvy is seen in babies fed cow’s milk due to the pasteurization process that denature the ascorbate [1, 2].

Despite its rarity, recent reports have highlighted cases of scurvy in children with neurological pathology and poorly adapted diet [9]. A review of the English medical literature made by Harknett *et al.* showed 18 cases of scurvy in pediatrics patients who had been diagnosed with autism and neuro-developmental delay with limited food preferences [1]. The study made by Noble *et al.* [10] has shown 23 cases of scurvy in children with behavioral disorders, including children with autism and children with cerebral palsy. The initial manifestations are non-specific such as irritability, loss of appetite, low grade fever and later dermatological such as petechiae, ecchymoses, hyperkeratosis and cork screw hairs [11].

Then, chronic bleeding is seen in areas where the blood vessels are superficial or areas where the contraction of muscles is enough to traumatize already defective blood vessels’s wall. The major systemic signs of scurvy in children include fatigue, weight curve decay, loss of appetite and anxiety [2]. Biological findings are not specific. Anemia is frequent, and may be hypochromic, normocytic, or macrocytic. Although, bleeding may contribute to induce anemia, the main factor is concomitant iron and folic acid deficiency.

**Fig. (6):**
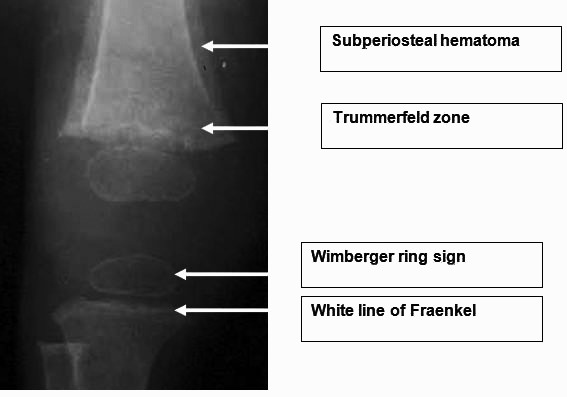
Radiological sings of Scurvy.

Musculoskeletal abnormalities are found in 80% of patients with scurvy [1, 3, 4, 9]. These symptoms are seen later and consist of joint’s pain and swelling, as well as myalgia, progressive muscular hypotrophy and unexplained fractures. The radiographic findings of infantile scurvy are not usually present. The most specific are: osteoporosis of the epiphysis which is surrounded by a white line of calcification called ring sign or Wimberger sign; epiphyseal separations; transverse line of increased density called white line or Frankel line and a transverse metaphyseal bands of decreased density in the side of the last one called scurvy line or trùmmerfeld zone [12] (Fig. **6**).

MRI findings show a diffuse multifocal decreased signal on T1- weighted imaging and increased signal on T2-weighted imaging within bone marrow, predominantly within the metaphyses, a varying degree of bone marrow enhancement, and adjacent periosteal elevation and soft tissue signal abnormalities [13, 14]. A study by Duggan *et al.* showed a much more notable increase in the amount of subperiosteal hematoma; hence the recurrent subperiosteal hematoma was an important clue for the diagnosis of scurvy [15].

The definite diagnosis is achieved by determining the serum ascorbic acid level. A low plasma level of vitamin C (<0.2 mg/dl) is specific in scurvy. However, the levels may be normal if there has been recent vitamin C supplementation in any form. Thus, determination of plasma vitamin C levels remains an insensitive laboratory test for vitamin C deficiency. Measuring the vitamin C level in the buffy-coat of the leucocytes is a better estimate of the vitamin body stores. However, this method is technically demanding and not always available [6].

Due to its multiple differential diagnoses (Table **1**), scurvy is difficult to diagnose, but there is a useful mnemonic for remembering many of its common presentations is 4 “H”: hemorrhagic signs, hyperkeratosis, hematologic abnormalities, and hypochondriasis [6].

## CONCLUSION

Scurvy can occur in each era despite the availability of alimentary sources of vitamin C.

It is rare in children; however, its presentation among risky populations should not be forgotten.

Musculoskeletal revelations, mostly subperiosteal hematoma, are the main manifestation of scurvy in the pediatric population.

The diagnosis of scurvy is based on clinical and radiological findings and low serum vitamin C levels, while a good response of the patient to vitamin C treatment makes the diagnosis definitive.

In risky children, scurvy should be prevented by systematic dietary supplementation of vitamin C.

A heightened awareness is needed to avoid an unnecessary surgery, unnecessary tests and procedures and to be able to start treatment for a potentially fatal but easily curable disease.

## Figures and Tables

**Table 1:** Common differential diagnosis of scurvy.

1. Osteomyelitis 2. Septic arthritis 3. Vitamin D deficiency 4. Child abuse and neglect 5. Thrombophlebitis 6. Leukemia 7. Pediatric syphilis 8. Platelet dysfunction

## LIST OF ABBREVIATIONS

µL: Microliter
g/dl: Gram per deciliter
mg/dl: Milligram per deciliter
mg: Milligram
mm Hg: Millimeter of mercury
mm/h: Millimeter per hour
MRI: Magnetic resonance imaging
